# A 2-year RSA study of the Vanguard CR total knee system: A randomized controlled trial comparing patient-specific positioning guides with conventional technique

**DOI:** 10.1080/17453674.2018.1470866

**Published:** 2018-05-09

**Authors:** Frank-David Øhrn, Justin Van Leeuwen, Masako Tsukanaka, Stephan M Röhrl

**Affiliations:** 1 Kristiansund Hospital, Møre and Romsdal Health Trust, Kristiansund;; 2 NTNU Norwegian University of Science and Technology;; 3 Department of Orthopaedic Surgery, Betanien Hospital, Skien;; 4 Division of Orthopaedic Surgery, Oslo University Hospital, Oslo;; 5 Institute of Clinical Medicine, Faculty of Medicine, University of Oslo, Norway

## Abstract

**Background and purpose** — There is some concern regarding the revision rate of the Vanguard CR TKA in 1 registry, and the literature is ambiguous about the efficacy of patient-specific positioning guides (PSPGs). The objective of this study was to investigate the stability of the cemented Vanguard CR Total Knee using 2 different surgical techniques. Our hypothesis was that there is no difference in migration when implanting the Vanguard CR with either PSPGs or conventional technique. We hereby present a randomized controlled trial of 2-year follow-up with radiostereometric analysis (RSA).

**Patients and methods** — 40 TKAs were performed between 2011 and 2013 with either PSPGs or the conventional technique and 22 of these were investigated with RSA.

**Results** — The PSPG (8 knees) and the conventional (14 knees) groups had a mean maximum total point motion (MTPM) (95% CI) of 0.83 (0.48–1.18) vs. 0.70 (0.43–0.97) mm, 1.03 (0.60–1.43) vs. 0.86 (0.53–1.19), and 1.46 (1.07–1.85) vs. 0.80 (0.52–1.43) at 3, 12, and 24 months respectively (p = 0.1). 5 implants had either an MTPM >1.6 mm at 12 months and/or a migration of more than 0.2 mm between 1- and 2-year follow-ups. 2 of these also had a peripheral subsidence of more than 0.6 mm at 2 years.

**Interpretation** — 5 implants (3 in the PSPG group) were found to be at risk of later aseptic loosening. The PSPG group continuously migrated between 12 and 24 months. The conventional group had an initial high migration between postoperative and 3 months, but seemed more stable after 1 year. Although the difference was not statistically significant, we think the migration in the PSPG group is of some concern.

Not all patients are satisfied after total knee arthroplasty (TKA); in several studies up to 25% of patients have persistent pain and dysfunction (Baker et al. 2007, Beswick et al. 2012, Howells et al. 2016). Many revisions are caused because of aseptic loosening of the implant. Younger patients undergoing TKA (Kurtz et al. 2009, Ravi et al. 2012) show a higher revision rate (Civinini et al. 2017). Thus, patient dissatisfaction, aseptic loosening, and demographic changes are good reasons to try to improve prosthesis designs and surgical precision. At the same time, all changes in clinical practice or choice of implant should follow the principle of stepwise introduction (Malchau 2000, Nelissen et al. 2011, Pijls and Nelissen 2016).

The Vanguard Cruciate Retaining (CR) Total Knee (Vanguard Complete Knee System, Zimmer Biomet Inc., Warsaw, IN, USA) was introduced in 2003. In some registries (AOANJRR, NJR) the prosthesis has showed promising results. Yet in another (SKAR), the Vanguard CR had a significantly higher relative risk of revision compared with other implants.

The implant can be inserted by conventional surgical technique or with patient-specific positioning guides (PSPGs). PSPGs are customized and manufactured from preoperative CT or MRI data to improve postoperative alignment (van Leeuwen et al. 2015). The literature is still ambiguous regarding the efficacy of PSPGs (Boonen et al. 2012, Nunley et al. 2012, An et al. 2017). Altered surgical technique or alignment might influence the early stability of the implants.

The hypothesis of this study was that the cemented Vanguard CR TKA is a stable implant using PSPGs. Therefore we investigated the stability of the cemented Vanguard CR Total Knee using 2 different surgical techniques.

## Patients and methods

This study was part of a randomized controlled multicentre trial (RCT) in Oslo and Skien, Norway, which compared clinical and radiological but no radiostereometric analysis (RSA) results of the PSPG technique (Signature Personalized Patient Care System; Zimmer Biomet) with the conventional technique for TKA. The exclusion criteria were published in that study (van Leeuwen et al. 2018). All surgeries and investigations in the RSA cohort were performed at Ullevål Hospital, Oslo, Norway.

**Figure 1.**
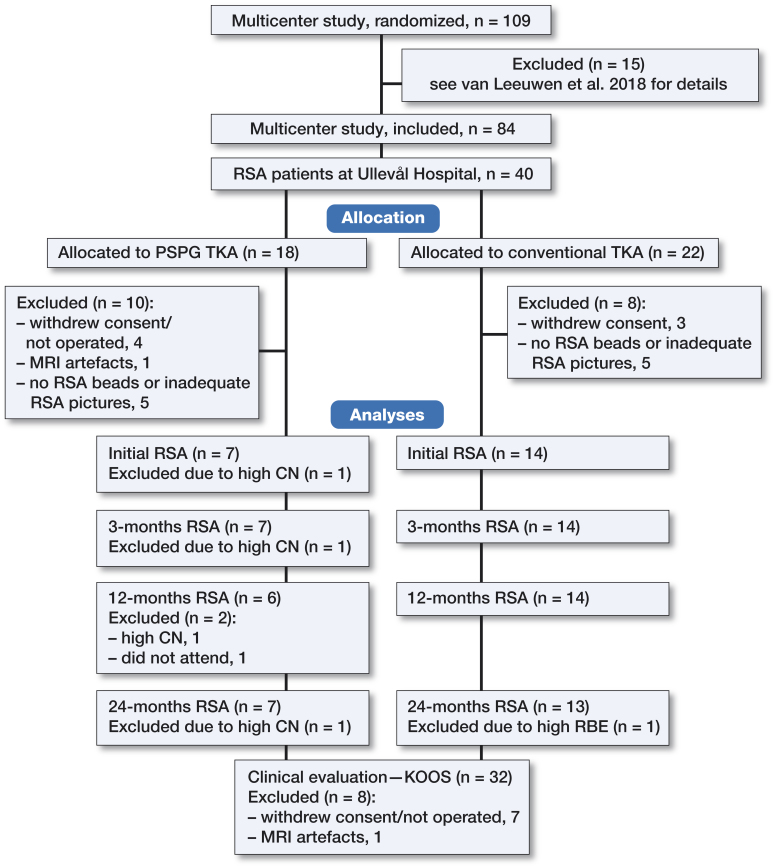
Flow chart.

40 patients participated in the RSA study at Ullevål Hospital, but only 22 were included in the RSA analyses (Figure 1). These were operated between December 2011 and December 2013. A Vanguard Cruciate Retaining (CR) TKA was performed in all patients (Cemented Vanguard Complete Knee System; Zimmer Biomet Inc., Warsaw, IN): 8 with the PSPG technique and 14 with the conventional surgical method.

### Design

This study was designed as a single blinded RCT of patients receiving TKA for symptomatic osteoarthritis of the knee.

### Patients

The participants were assigned to either the conventional or PSPG technique according to the protocol of the multicentre RCT with block randomization obtained by variable block sizes (van Leeuwen et al. 2018). In the original RCT the sample size was calculated for the frontal mechanical axis and the secondary outcome KOOS score. That study was terminated when the total number of patients was sufficient according to the primary outcome measure, hence the suboptimal number of patients in the RSA study (Figure 1). The surgeries were performed by 2 experienced surgeons. 7 patients withdrew their consent or were not operated for various reasons after randomization, 1 had MRI artefacts that precluded manufacturing of PSPGs, and 10 did not have beads inserted or had inadequate RSA pictures, thus only 22 had RSA in this study. At 2 of the time points there were only 20 patients included in these analyses. 1 patient had a high condition number (CN) and was excluded at all time points, 1 did not show up at 12 months, and 1 had high rigid body error (RBE) at 24 months.

### Intervention

We used a standard midline incision and medial parapatellar capsulotomy in all patients. A tourniquet was used in all cases. For details of the operative procedure see van Leeuwen (2018). Both surgical techniques (conventional and PSPG) for this implant were well established in the department prior to the inclusion of RSA patients, so we assumed that there was no learning curve.

During surgery 6 to 8 1.0 mm tantalum markers (RSA Biomedical, Umeå, Sweden) were inserted in the tibia. All patients followed the same standardized postoperative rehabilitation protocol.

### Evaluation

Implant migration was evaluated using RSA. The first examination took place within a week postoperatively, then after 3, 12, and 24 months. They were all performed in the supine position by the same radiographers at each time point. We used calibration cage number 43 (RSA Biomedical, Umeå, Sweden) and ceiling mounted X-ray tubes (Proteus XR/A, GE Healthcare and Canon Triathlon T3).

**Figure 2.**
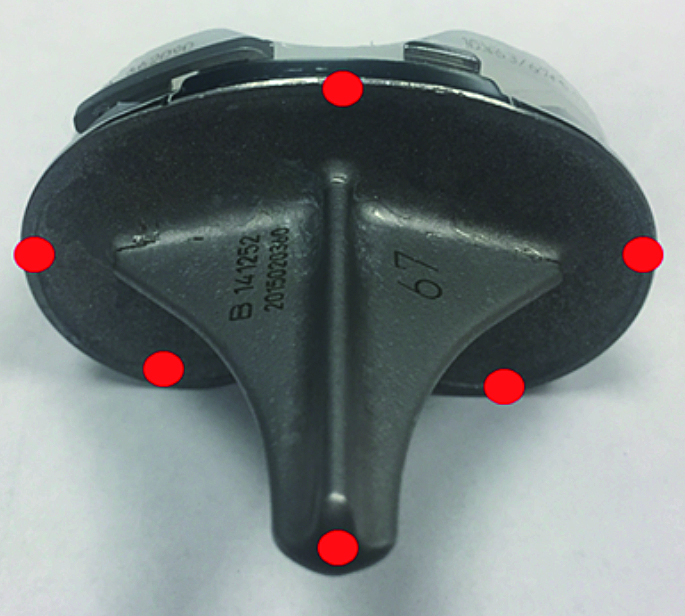
Fictive points of the tibial implant (the posterior fictive point is hidden behind the stem).

MB-RSA 3.40 (RSAcore, Leiden, The Netherlands) software was used for the migration analysis. The migration was described both as segment motion of all 6 degrees of freedom (translations and rotations) and maximum total point motion (MTPM), the latter being primary outcome. In addition, we analyzed the point motion from fictive points added to the computer aided design (CAD) model of the tibial component. We had the following 7 fictive points: stem tip, anterior, posterior, posteromedial, posterolateral, medial, and lateral (Figure 2). All these points were reported for X, Y, and Z translations. As we performed double examinations, the 2 RSA pictures were run against all the others, making a total of 4 motions for each patient at each time point. The average of these 4-point motions represented the motion of the individual implant at each time point. The movements of the 13 left knees were converted to right knees for stability analysis (Valstar et al. 2005).

Our upper limit for CN was 100, and for RBE 0.50 mm. 1 patient exceeded 0.5 mm RBE at 2 years and was excluded from the RSA examination at this time point. The rest had RBE of less than 0.35mm. Precision was assessed by double RSA examinations of all patients at all time points, and reported as absolute mean difference of double examinations ±1.96 x standard deviation (SD).

For clinical assessment we used the Knee injury and Osteoarthritis Outcome Score (KOOS) (Roos et al. 1998). All complications were registered.

### Statistics

We used linear mixed models to evaluate differences in MTPM, translations, rotations, and point motions within groups (PSPG vs. conventional) over the entire follow-up period and to control for repeated measurements. The fixed effects were time, group, and time-by-group interaction. The model included a random slope. As the KOOS scores were not normally distributed, the Wilcoxon signed-rank test was used to evaluate difference from preoperative to 2 years. Further, patients were divided into high- and low-risk group according their migration data (Ryd et al. 1995, Pijls et al. 2012). To estimate differences in KOOS scores between these 2 independent groups we used the Mann–Whitney U test. Fisher’s exact test was used to detect associations between categorical independent variables.

The results are reported as means or proportions with 95% confidence intervals (CI), if not stated otherwise.

All statistical calculations were performed using the IBM SPSS Statistics version 23 (IBM Corp, Armonk, NY, USA).

### Ethics, registration, funding, and potential conflicts of interest

The study was approved by the Regional Committee for Medical and Health Research Ethics, West-Norway (REC West, approval number 2010/2056) and the institutional review board at Oslo University Hospital (2011/7613), and registered at clinicaltrials.gov (NCT01696552). All patients were included with written consent. No financial funding from companies has been received for this study, and the authors declare that there are no conflicts of interest.

## Results

### Demographics

See Table 1 for baseline characteristics.

**Table 1.** Baseline characteristics of conventional vs. PSPG patients. Values are mean (SD) (range) unless otherwise specified

Factor	Conventional	PSPG
Number of patients	14	8
Left/right, n	10/4	3/5
Men/women ratio, n	7/7	2/6
Age	65 (7.9) (53–77)	60 (5.1) (50–68)
BMI	28 (4.2) (22–36)	30 (4.8) (22–35)
Body weight (kg)	84 (18) (60–105)	87 (12) (70–100)
Operation time (min)	121 (37) (68–228)	111 (8.9) (95–125)
Postoperative HKA (°)	181 (4.8) (172–188)	178 (5.7) (171–186)

### RSA

**Figure 3.**
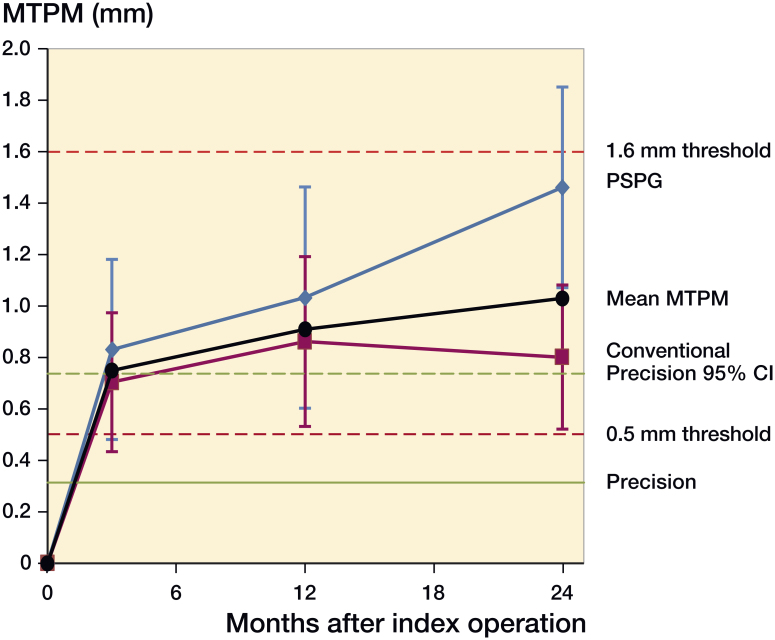
Mean MTPM over time for the whole cohort and for the PSPG and conventional groups with thresholds (Pijls et al. 2012).

**Figure 4.**
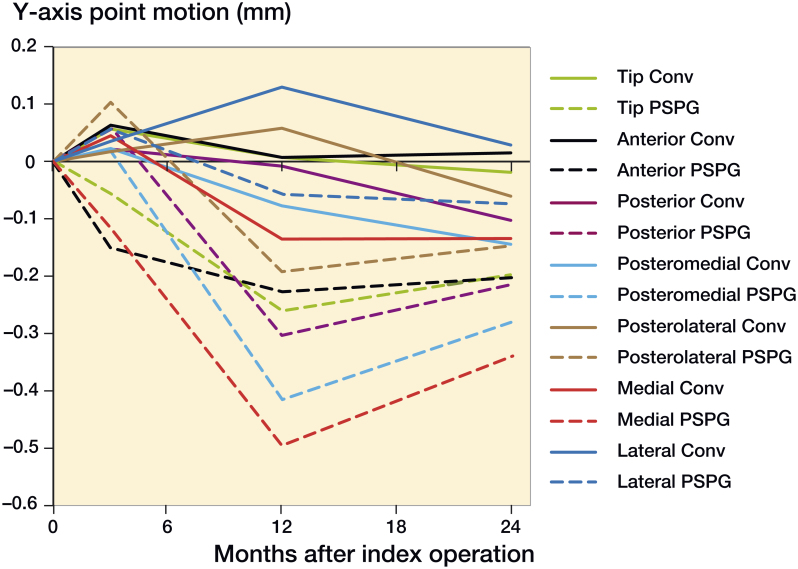
Y (axial, lift-off, subsidence) point motions stratified in PSPG (dashed lines) vs. conventional.

**Figure 5.**
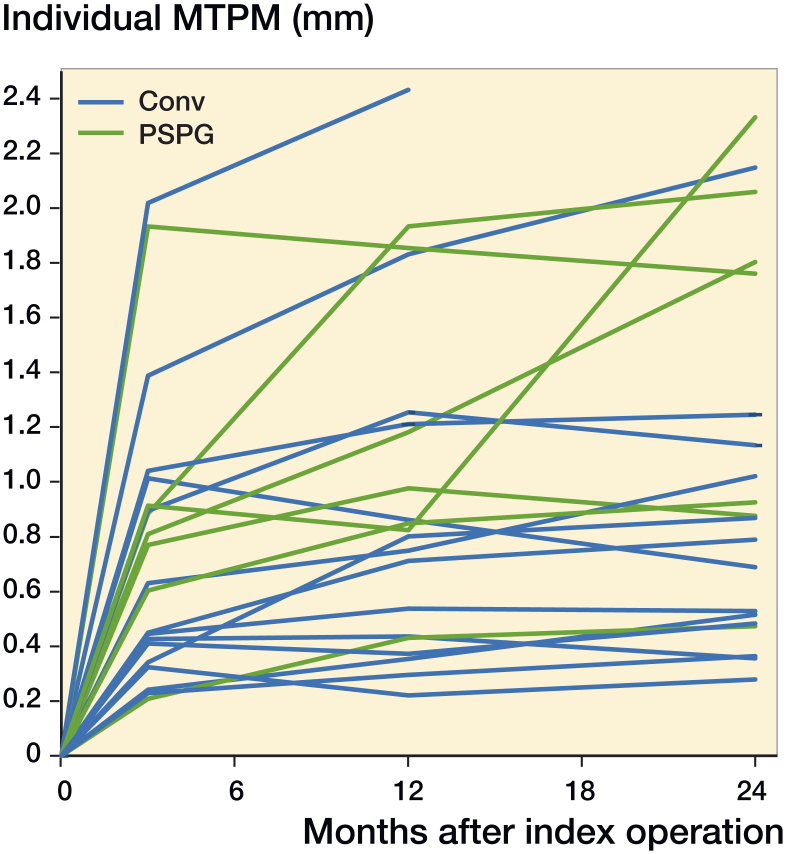
Individual time profiles of MTPM in the two subgroups (n = 21). Conventional marked with blue lines, PSPGs with green lines.

**Figure 6.**
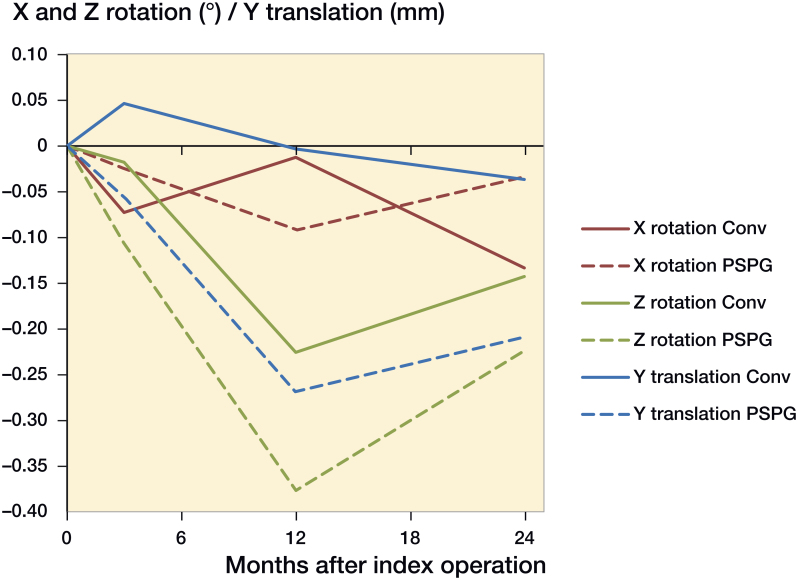
X and Z rotation in degrees and Y translation in mms (PSPG vs. conventional).

The mean MTPM, and relevant point motions, translations, and rotations are shown in Tables 2 and 3 (see Supplementary data) and Figures 3–6. The PSPG group had an increasing migration pattern compared with the conventional group. The results from the linear mixed model analysis showed a statistically significant change in MTPM within (p < 0.001), but not between the 2 groups after 2 years (p = 0.1) (Table 4). Generally in the point motions analysis, we found a larger subsidence in the PSPG than the conventional group, but no statistical significance could be found (Table 3, see Supplementary data, Figure 4).

**Table 4.** Results of the linear mixed model analysis of MTPM at 2 years after randomization into PSPGs and conventional subgroups

Parameter	Coefficient	p-value	95% CI
Intercept	–0.13	0.2	–0.32 to 0.07
Time	0.30	< 0.001	0.18 to 0.42
Randomization	–0.18	0.3	–0.50 to 0.14
Time x Randomization	0.16	0.1	–0.04 to 0.36

On an individual basis 4 implants had more than 1.6 mm migration at 12 months, but 1 of these was excluded due to an RBE >0.5 mm at 2 years (Figure 5). 4 implants had more than 0.2 mm migration between 1- and 2-year follow-ups. Of the 3 remaining patients with MTPM >1.6 mm at 1 year, 2 had migration of more than 0.2 mm between 1 and 2 years, hence 5 patients had either >1.6 mm at 1 year, or >0.2 mm migration between 1 and 2 years. 2 of these patients also met the criteria for distal or proximal peripheral translation. No other implants met these criteria. None of the implants met the criteria of transversal rotation (Gudnason et al. 2017). In 3 of these 5 high-risk patients the PSPG method had been used.

The precisions of our RSA examinations were the following: 0.31 mm for MTPM (95% CI 0.00–0.74), 0.01 mm for X translation (95% CI –0.12 to 0.14), 0.01 mm for Y translation (95% CI –0.07 to 0.08), 0.03mm for Z translation (95% CI –0.25 to 0.32), 0.05° for X rotation (95% CI –0.28 to 0.37), 0.04° for Y rotation (95% CI –0.65 to 0.72), and 0.00° for Z rotation (95% CI –0.14 to 0.15).

### Clinical results

We found a statistically significant improvement of all the KOOS subscales from preoperative through 2 years in the whole cohort. We could not see any difference in clinical performance for implants with migration at risk or PSPG and conventional groups (Mann–Whitney U test) (Figure 7, see Supplementary data).

Neither could we demonstrate any other subgroup to explain the inferior stability of the high-risk group (Table 5, see Supplementary data).

Among the patients with high-risk migration we found 1 with inferior clinical scoring. This person was in the conventional group and had a postoperative hematoma with no need for further surgery. Postoperative radiographs showed an HKA-angle of 172° (varus).

### Complications

5 complications occurred: 1 deep hematoma that was evacuated, 1 stiff knee requiring mobilization under anaesthesia, 2 superficial hematomas, and 1 superficial infection. The latter was in the PSPG group, the others in the conventional group. None of the complications required reoperation. Except for the aforementioned high-risk patient, they had good clinical scores after 2 years.

## Discussion

Our main finding was that the implants in the PSPG group had continuous migration between 12 and 24 months. The implants in the conventional group showed migration between postoperatively and 3 months; thereafter the mean migration abated and the implant stabilized. The difference in MTPM and subsidence between the two groups, however, was not statistically significant.

Several studies have discussed threshold levels for increased risk of aseptic loosening. Ryd et al. (1995) showed that the process of loosening probably starts directly after the operation and that a migration of more than 0.2 mm after 1 year gives a high risk of revision. Pijls et al. (2012) showed that more than 1.6 mm migration at 12 months gives an “unacceptable” risk of later revision. Gudnason et al. (2017) recently suggested a transversal rotation of more than 0.8°, or peripheral distal or proximal translation of more than 0.6 mm or 0.9 mm respectively at 2 years as a threshold. 5 of the 22 implants in our study met 1 or more of these criteria for high-risk implants. We could not identify any other factor than surgical technique that could explain why these knees performed worse than the rest, such as obesity, age, postoperative valgus or varus. However, due to the limited numbers of observations, emphasis should be given to the estimated values rather than p-values. All the high-risk patients, except for 1, also performed well clinically, with no symptoms of early loosening after 2 years. It is important to stress that although implants for individual patients met the criteria for “unacceptable risk” according to Pijls et al. (2012), it does not mean that the specific implant is loose. The meta-analysis of Pijls focused on mean MTPM with 12 months’ observation time, mainly because not all studies reported 2-year results, or migration in all degrees of freedom. The discussion concerning which criteria to follow therefore continues. In our study, as many as 5 of 22 patients were at risk of later loosening based on several studies (Ryd et al. 1995, Pijls et al. 2012), but following the recent study by Gudnason et al. (2017), only 2 implants were at risk of aseptic loosening, 1 in each group. Also, in that study the authors concluded that MTPM after 1 and 2 years is inferior to transversal rotation, peripheral subsidence, and lift-off in predicting late aseptic loosening.

A limitation of our study is the sample size. Ideally, we would have liked between 25 and 30 participants in each group, yet several other RSA studies have suboptimal sample sizes for various reasons (Hansson et al. 2005, Molt and Toksvig-Larsen 2014, Henricson and Nilsson 2016, Meinardi et al. 2016). RSA research is costly and tedious work, and it is not always possible to recruit enough patients. As the cohort was part of a larger RCT assessing clinical and radiological outcome of 2 different surgical methods, the RSA study was not powered as an RCT. This may be a possible reason why we could not find a statistically significant difference in MTPM between the 2 groups. In addition, in the larger RCT, a statistically significant difference was found in the position of the tibia in the frontal and sagittal planes (van Leeuwen et al. 2018). As the surgical techniques were well established in the department, we assume there was no learning curve. Longer follow-up of both groups is needed, especially with a focus on the continuous migration of the PSPG group.

One strength of our study is that we used fictive points in our RSA model. We could therefore show with which pattern the implant was migrating. Many studies include only MTPM and the segmental micromotions, their absolute values are often smaller than the peripheral point motions, and they do not tell us exactly how the implant migrates. Thus we could also evaluate the implant with respect to Gudnason’s data (Gudnason et al. 2017).

The long-term results of the implant we used diverge in the literature. Some registries show excellent results after 5- and 10-year follow-up (AOANJRR, NJR), the latter with only a few hundred patients reaching 10 years, and with no information regarding surgical technique. Several clinical studies also show excellent results (Kievit et al. 2014, Schroer et al. 2014, Faris et al. 2015, Emerson et al. 2016, Flament et al. 2016). To our knowledge, there is only 1 other study that has assessed the Cemented Vanguard CR with RSA (Schotanus et al. 2017). They found a mean MTPM for this implant of 0.7 mm at 12 months and 0.8 mm after 24 months. Although slightly lower migration than our data suggest, it leaves the implant in the same risk category according to Pijls et al.(2012). However, they did not use PSPGs. Another register found the implant to perform worse compared with other implants (SKAR). This effect is not present when a patellar button is implanted during primary surgery. As long-term data are still lacking, especially on the Signature System (PSPG), and only 1 RSA study shows early follow-up data on the implant, our study adds knowledge for users of this implant.

In summary we found that the cemented Vanguard CR had a higher initial mean migration than expected at 12 months, but from 12–24 months the conventional group stabilized. The PSPG group also had continuous migration at this point. None of the implants in our study rotated more than recommended, and only 2 implants had a total peripheral subsidence above that recommended, 1 in each group. Although the PSPG group did not have a statistically different MTPM from the conventional group, we think that the findings of the migration pattern of this technique are of some concern and call for longer follow-up.

### Supplementary data

Tables 2, 3, and 5 and Figure 7 are available in the online version of this article, http://dx.doi.org/10.1080/17453674.2018.1470866

FDØ analysed parts of the data and wrote the manuscript. MT analysed parts of the data and critically reviewed the manuscript. JvL and SMR designed the study and critically reviewed the manuscript.

The authors would like to thank the radiographers Mona Risdal, Silje Clausen, and Alexis Hinojosa, statistician Cathrine Brunborg and the study coordinators Marte Traae Magnusson and Anette Simonsen for their contributions to the study.

*Acta* thanks Bart G Pijls and Nikolaj Sebastian Winther for help with peer review of this study.

## Supplementary Material

IORT_A_1470866_SUPP.pdf
