# Supplementary material for: A 2-year RSA study of the Vanguard CR total knee system: A randomized controlled trial comparing patient-specific positioning guides with conventional technique
Source: Acta Orthop. 2018 May 9;89(4):418–24. doi: 10.1080/17453674.2018.1470866 (PMC6066770; doi:10.1080/17453674.2018.1470866)
Supplement: IORT_A_1470866_SUPP.pdf [file IORT_A_1470866_SM8920.pdf]

Supplementary data

Table 2. Mean MTPM (95% CI)

	3 months	12 months	24 months
Conv.	0.70 (0.43–0.97)	0.86 (0.53–1.19)	0.80 (0.52–1.08)
PSPG	0.83 (0.48–1.18)	1.03 (0.60–1.43)	1.46 (1.07–1.85) [a]
Diff. [b]	0.13 (0.05–0.21)	0.17 (0.07–0.24)	0.66 (0.55–0.77)

[a] P-value (mixed model analysis) = 0.1
[b] Difference of the mean MTPM (PSPG – conventional group) (CI).

Table 3. Point motions (mm) (95% CI) at 24 months

Fictive points	Translations at 24 months X	Y	Z
Tip			
Conv.	–0.07 (–0.16 to 0.02)	–0.02 (–0.17 to 0.13)	0.17 (0.00 to 0.34)
PSPG	–0.27 (–0.54 to 0.01)	–0.20 (–0.51 to 0.12)	0.22 (–0.15 to 0.58)
Anterior			
Conv.	0.05 (–0.06 to 0.15)	0.02 (–0.11 to 0.14)	0.05 (–0.14 to 0.24)
PSPG	–0.14 (–0.40 to 0.12)	–0.20 (–0.48 to 0.07)	0.19 (–0.57 to 0.94)
Posterior			
Conv.	0.18 (–0.10 to 0.46)	–0.10 (–0.37 to 0.16)	0.10 (–0.09 to 0.29)
PSPG	–0.23 (–0.78 to 0.33)	–0.21 (–0.88 to 0.45)	0.19 (–0.56 to 0.93)
Posteromedial			
Conv.	0.17 (–0.10 to 0.43)	–0.14 (–0.44 to 0.15)	0.20 (–0.04 to 0.43)
PSPG	–0.22 (–0.76 to 0.32)	–0.28 (–1.08 to 0.53)	0.11 (–0.78 to 0.99)
Posterolateral			
Conv.	0.19 (–0.10 to 0.48)	–0.06 (–0.31 to 0.19)	0.01 (–0.20 to 0.21)
PSPG	–0.23 (–0.78 to 0.33)	–0.15 (–0.72 to 0.42)	0.26 (–0.38 to 0.91)
Medial			
Conv.	0.06 (–0.11 to 0.23)	–0.13 (–0.40 to 0.13)	0.27 (–0.03 to 0.56)
PSPG	–0.14 (–0.48 to 0.21)	–0.34 (–1.12 to 0.45)	0.03 (–1.01 to 1.08)
Lateral			
Conv.	0.11 (–0.10 to 0.32)	0.03 (–0.17 to 0.22)	–0.11 (–0.39 to 0.17)
PSPG	–0.15 (–0.54 to 0.24)	–0.07 (–0.39 to 0.25)	0.34 (–0.27 to 0.94)

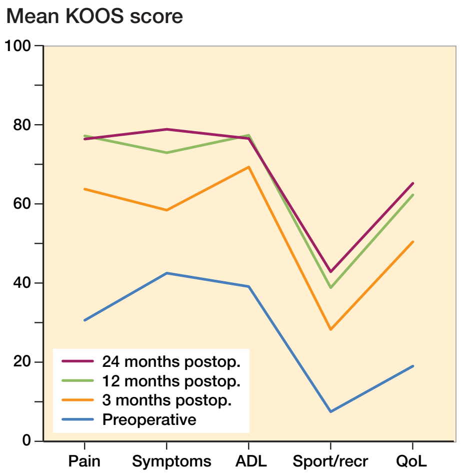

Figure 7. Knee injury and Osteoarthritis Outcome Score (KOOS) of the whole cohort (n = 32).

Table 5. Different variables from patients stratified by high versus low risk of aseptic loosening. Values are mean (95% CI) unless otherwise specified

Variable	Low risk (n = 16)	High risk (n = 6)	Y translation (or p-value)	Low risk (n = 16)	High risk [e] (n = 5)
BMI	28 (26–31)	30 (26–33)	Tip Y	0.0 (–0.1 to 0.7)	–0.3 (–0.9 to 0.3)
Weight (kg)	85 (76–93)	87 (72–101)	Anterior Y	0.0 (–0.1 to 0.1)	–0.2 (–0.7 to 0.3)
Operating time (min)	118 (100–136)	113 (100–127)	Medial Y	–0.2 (–0.4 to 0.0)	–0.3 (–1.7 to 1.0)
Postop. HKA (°)	181 (178–183)	177 (171–183)	Posteromedial Y	–0.1 (–0.3 to 0.1)	–0.5 (–1.9 to 0.9)
Tibia frontal p.o. CT (°) [c]	–0.3 (–1.2 to 0.6)	2.1 (–1.2 to 5.3)	Lateral Y	0.1 (0.0 to 0.2)	–0.4 (–0.9 to 0.0)
Tibia sagittal p.o. CT (°) [c]	–5.8 (–7.7 to –3.9)	–5.3 (–8.1 to –2.4)	Posterolateral Y	0.1 (–0.1 to 0.2)	–0.6 (–1.5 to 0.4)
Age (years)	62 (59–65)	66 (56–76)	Posterior Y	0.0 (–0.2 to 0.1)	–0.5 (–1.7 to 0.6)
			MTPM 0–12 months (mm)	0.7 (0.5 to 0.9)	1.5 (0.8 to 2.2)
			MTPM 12–24 months (mm)	0.0 (0.0 to 0.1)	0.5 (–0.1 to 1.1)
Male gender	5 (31%)	4 (67%)	p-value [a] 0.2		
Tibia sagittal outliers [b,c]	9 (60%) [d]	3 (60 %) [d]	p-value [a] 1		
Tibia frontal outliers [b,c]	0 (0%) [d]	1 (20 %) [d]	p-value [a] 0.3		

[a] Fisher's exact test.
[b] Outlier > 3°
[c] 2 patients were excluded from CT evaluation due to wrong protocol, 1 of them a high-risk implant (total n = 20, high risk n = 5).
[d] Percentage within risk profile.
[e] 1 patient excluded due to high RBE.
